# Effect of the *S*‐nitrosoglutathione reductase inhibitor N6022 on bronchial hyperreactivity in asthma

**DOI:** 10.1002/iid3.220

**Published:** 2018-04-11

**Authors:** Loretta G. Que, Zhonghui Yang, Njira L. Lugogo, Rohit K. Katial, Steven A. Shoemaker, Janice M. Troha, David M. Rodman, Robert M. Tighe, Monica Kraft

**Affiliations:** ^1^ Departmentof Medicine Duke University Health System Durham North Carolina USA; ^2^ Division of Allergy & Clinical Immunology National Jewish Health Denver Colorado USA; ^3^ Nivalis Therapeutics Inc. Boulder Colorado USA; ^4^ Department of Medicine College of Medicine University of Arizona Health Sciences Tucson Arizona USA

**Keywords:** mild asthma, N6022, *S*‐nitrosoglutathione, *S*‐nitrosoglutathione reductase

## Abstract

**Rationale:**

Patients with asthma demonstrate depletion of the endogenous bronchodilator *GSNO* and upregulation of *GSNOR*.

**Objectives:**

An exploratory proof of concept clinical study of N6022 in mild asthma to determine the potential bronchoprotective effects of *GSNOR* inhibition. Mechanistic studies aimed to provide translational evidence of effect.

**Methods:**

Fourteen mild asthma patients were treated with intravenous N6022 (5 mg) or placebo and observed for 7 days, with repeated assessments of the provocative dose of methacholine causing a 20% fall in FEV1 (methacholine PC_20_ FEV1), followed by a washout period and crossover treatment and observation. In vitro studies in isolated eosinophils investigated the effect of *GSNO* and N6022 on apoptosis.

**Measurements and Main Results:**

This was a negative trial as it failed to reach its primary endpoint, which was change from baseline in methacholine PC_20_ FEV1 at 24 h. However, our exploratory analysis demonstrated significantly more two dose‐doubling increases in PC_20_ FEV1 for N6022 compared with placebo (21% vs 6%, *P *< 0.05) over the 7‐day observation period. Furthermore, a significant treatment effect was observed in the change in PC_20_FEV1 from baseline averaged over the 7‐day observation period (mean change: +0.82 mg/ml [N6022] from 1.34 mg/ml [baseline] vs −0.18 mg/ml [placebo] from 1.16 mg/ml [baseline], *P* = 0.023). N6022 was well tolerated in mild asthmatics. In vitro studies demonstrated enhanced eosinophilic apoptosis with N6022.

**Conclusions:**

In this early phase exploratory proof of concept trial in asthma, N6022 did not significantly alter methacholine PC_20_ FEV1 at 24 h, but did have a treatment effect at 7 days compared to baseline. Further investigation of the efficacy of *S*‐nitrosoglutathione reductase inhibition in a patient population with eosinophilic asthma is warranted.

## Background

Asthma is defined as a chronic inflammatory disorder of the airways, which is characterized by variable and recurring respiratory symptoms, airflow limitation, or obstruction, and airway hyperresponsiveness (AHR) 1. Exhaled nitric oxide (NO) is high in a proportion of patients with severe asthma 2 typically those with type 2 driven inflammation characterized by the increased concentrations of cytokines IL‐4, IL‐5, and IL‐13, serum IgE, and blood and sputum eosinophils 3. *S*‐Nitrosothiols (SNOs) such as *S*‐nitrosoglutathione (GSNO), an endogenous bronchodilator, are considered to be integral to the physiological functioning of NO 4, 5 and SNO metabolism is reported to govern NO‐related bioreactivity in the airways 5. Increases in NO are thought to be beneficial when channeled into SNOs, and the main determinant of whether NO synthase (NOS) activity impacts asthma may be related to the extent of preservation of SNO‐based signaling 5. GSNO, the major source of NO bioactivity in the lung, is reduced in asthma and Que et al. 5 demonstrated that by maintaining GSNO, animals are protected from asthma. Through their impact on bronchial smooth muscle tone and responsivity, adrenergic receptor function, and anti‐inflammatory activities, NO and GSNO help to maintain normal lung physiology and function 5, 6, 7, 8.

GSNO reductase (GSNOR) is a key regulator of GSNO in the lung where it acts to metabolize GSNO to oxidized glutathione and ammonia. Studies have demonstrated that the concentration of SNOs is lower in asthmatic versus non‐asthmatic lungs 9, 10. In addition, we reported that the expression and activity of GSNOR is increased in asthma 11. Evidence suggests that inhibition of GSNOR can lead to preservation of endogenous GSNO. Indeed, polymorphisms in GSNOR increase GSNOR expression and are associated with increased risk of asthma and lower beta‐agonist responsiveness 12, 13, 14. In addition, mouse models of allergic asthma have shown that the loss of GSNOR protects against AHR 5, and that GSNOR inhibition limits eosinophilic inflammation, mucus production, and AHR 15. Thus, GSNOR inhibition may offer a novel therapeutic strategy in asthma with a type 2 inflammatory phenotype.

N6022 is a potent and reversible small‐molecule inhibitor of GSNOR with a half‐maximal inhibitory concentration (IC_50_) of 8 nM and an inhibitory constant (K_i_) of 2.5 nM 16. It has been found to protect wild‐type, ovalbumin‐sensitized and challenged mice from methacholine‐induced AHR while significantly decreasing eosinophilic infiltration and inflammatory biomarkers in bronchoalveolar lavage fluid compared with controls 17. GSNOR inhibitors, such as N6022, represent a new class of therapy with the potential to treat asthma through a combination of bronchodilatory and anti‐inflammatory effects. The precise mechanism by which enhanced GSNO levels mediate an anti‐inflammatory effect is unknown, but it can be investigated using isolated eosinophils. Frequently observed in asthma, eosinophils are associated with higher disease burden 1. In recent years, therapeutics to reduce eosinophilia been developed, helping to improve clinical symptoms and lung function and to reduce exacerbations 18.

## Objective

Understanding the effect of GSNO and GSNOR inhibition on eosinophilic apoptosis will provide further insights into the anti‐inflammatory effect of GSNO levels in asthma. Here we present findings from an exploratory proof of concept clinical study aiming to determine the effect of the GSNOR inhibition with N6022 in mild asthma. A supporting mechanistic study aims to provide translational evidence of effect.

## Methods

### Clinical study design and assessments

**Figure 1:**
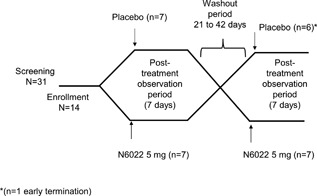
Study design and patient disposition.

An exploratory multicenter, randomized, double‐blind, placebo‐controlled crossover study was conducted. Fourteen eligible patients were enrolled at Duke University Medical Center (12 patients; Durham, NC) and National Jewish Hospital (2 patients; Denver, CO). Patients had a pre‐bronchodilator forced expiratory volume in 1 sec (FEV1) ≥75% predicted, a provocative concentration of methacholine (MCh) causing a 20% fall in FEV1 (MCh PC_20_ FEV1) of ≤8 mg/ml, and required less‐than‐daily use of short‐acting inhaled beta‐agonists. Patients were randomized to receive 5 mg N6022 IV or placebo and observed for 7 days, followed by a 21‐ to 42‐day washout period and subsequent crossover treatment (Fig. 1).

The selected dose of 5 mg N6022 was determined from efficacy 17 and safety studies in animal models of experimental allergic asthma and safety studies in healthy human subjects 19. Intravenous dosing of N6022 was utilized as the route of administration in this exploratory trial in an effort to eliminate delivery hurdles and to decrease PK variability that would most likely occur with inhaled drug delivery. Dosing was conducted on Day 1 of each treatment period. Patients were followed for efficacy, safety, and tolerability until discharge on the morning of Day 2 with follow‐up visits on Days 3 and 7. To evaluate the effect of N6022 on airways hyperresponsiveness, MCh challenge testing was performed at screening, at 8, 24, and 48 h post‐dose, and on Day 7. Repeated MCh challenge testing has been performed previously and has not been shown to induce tolerance to MCh in subjects with asthma 20. This study was conducted in compliance with the International Conference on Harmonisation Guidelines on Good Clinical Practice and was approved by the institutional review board at each institution prior to initiation; all patients provided written informed consent.

### Endpoints and assessments

The primary endpoint investigated whether a single dose of N6022 produced a significant bronchoprotective effect in patients with mild asthma, expressed as the MCh PC_20_FEV1 at 24 h post‐dose compared with placebo. Secondary endpoints investigated the bronchoprotective effect of N6022 at 8 h post‐dose, as well as the safety and tolerability of N6022.

Exploratory endpoints investigated the bronchoprotective effect of N6022 over 7 days (expressed as MCh PC_20_FEV1), and the effects of N6022 on serum eosinophil cationic protein (ECP) at 8, 24, and 48 h post‐dose, both compared with placebo. The impact of N6022 on inflammatory cell counts in induced sputum was examined at 24 h post‐dose as were respiratory symptoms using the Asthma Control Questionnaire (ACQ‐7). To evaluate the effect of N6022 on airways hyperresponsiveness, MCh challenge testing was performed at screening, at 8, 24, and 48 h post‐dose, and on Day 7 with early time points based on N6022 administration in animal models of asthma 17. Pharmacokinetic (PK) analyses of a single dose of N6022 were also performed.

### Statistics

The full set included all patients with an MCh PC_20_FEV1 at 24 h post‐dose during one of the treatment periods. A mixed‐effects model with fixed categorical effects for treatment, sequence, post‐dosing time point, and treatment‐by‐time‐point interaction was used. Correction for multiple comparisons were not performed in this exploratory study. Additional methods are provided in the Online Data Supplement.

### Mechanistic study design and assessments

To perform in vitro studies of the effect of GSNO and N6022 on eosinophil apoptosis, eosinophils were isolated from the whole blood of IL‐5–transgenic mice, cultured with 100, 250, or 500 μM GSNO, N6022, or dimethyl sulfoxide (DMSO) vehicle control for 20–24 h. At the end of incubation, eosinophils were washed thoroughly and stained using 7‐amino‐actinomycin D (7‐AAD) (BD Biosciences, San Jose, CA) and flow cytometry (fluorescence‐activated cell sorting; FACS) was performed on a FACS Canto II (BD Biosciences, San Jose, CA) to assess cell viability. To assess apoptosis, levels of cleaved caspase‐3 (R&D Systems, Minneapolis, MN) and β‐actin (Cell Signaling Technology, Danvers, MA) in whole blood eosinophils from IL‐5–transgenic mice were assessed by Western blot analysis.

See Online Data Supplement for additional information.

## Results

### Clinical research

#### Baseline characteristics

The study was conducted at two sites in the US, and all 14 patients randomized received N6022 (Fig. 1). All but one of the 14 patients also received placebo; one patient who received N6022 in the first treatment period withdrew from the study prior to the crossover, due to a lack of desire to remain in the study. Overall, the study population was predominantly Caucasian and male. The mean values for FEV1 and FEV1/forced vital capacity (FVC) were consistent with mild asthma (Table 1).

**Table 1:** Patient demographics and baseline characteristics (FAS; full analysis set population)

	Overall (*n *= 14)^*^
Female, *n* (%)	2 (14.3%)
Age, years mean (SD)	32.9 (12.4)
Race, *n* (%)	
Black/African American	2 (14.3)
White	11 (78.6)
Other	1 (7.1)
Ethnicity, *n* (%)	
Hispanic/Latino	1 (7.1)
BMI, kg/m^2^, mean (SD)	25.8 (2.2)
Weight, kg	79.3 (6.3)
Serum ECP, ng/mL, mean (SD)	21.6 (17.4)
Sputum inflammatory cell count, 10^4^ /mL, mean (SD)	153.1 (122.9)
Duration of asthma (years), mean (SD)	20.4 (10.1)
ACQ‐7 score, mean (SD)^†^	
N6022 (*n *= 14)	1.0 (0.6)
Placebo (*n *= 13)	0.9 (0.5)
Pulmonary function, mean (SD)	
FEV1 (L)	3.49 (0.45)
Percent Predicted FEV1 (%)	85.6 (6.08)
FEV1/FVC (%)	70.4 (6.64)
MCh PC_20_ FEV1, mg/ml, mean (SD)^*^	
N6022 (*n *= 14)	1.34 (2.00)
Placebo (*n *= 13)	1.16 (1.96)
Concommitant bronchodilators	
Albuterol	7 (50.0)

ACQ, asthma control questionnaire; BMI, body mass index; ECP, eosinophil cationic protein; FEV1, forced expiratory volume in 1 sec; FVC, forced vital capacity; MCh PC20 FEV1, the provocative concentration of methacholine causing a 20% fall in FEV1; SD, standard deviation.

^†^ The baseline for ACQ‐7 was determined on day –1 of each treatment period.

^*^ Fourteen patients were screened and received treatment with N6022. All but one of the 14 patients also received treatment with placebo; one patient who received N6022 in the first treatment period withdrew from the study prior to the crossover.

### Efficacy

#### MCh PC_20_FEV1

**Figure 2:**
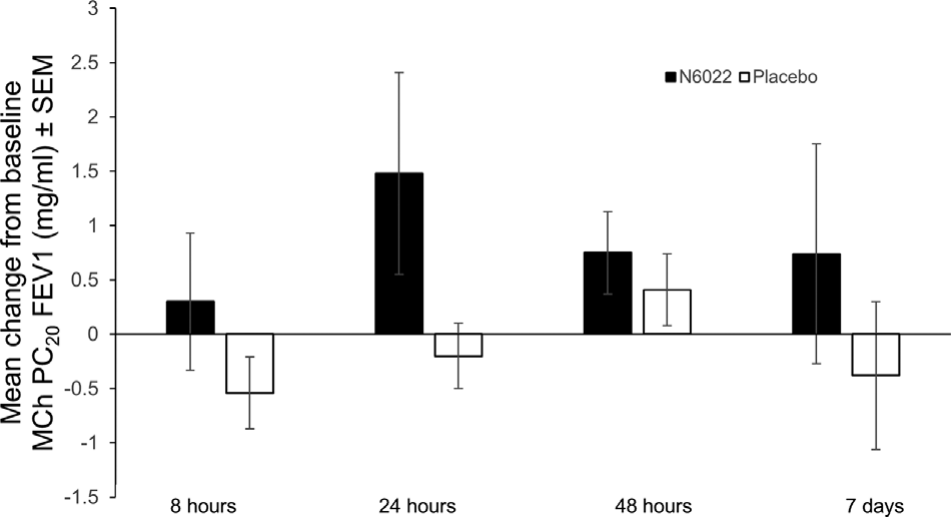
Mean change from baseline in MCh PC_20_ FEV1 increased over the N6022 post‐treatment observation period compared with the placebo observation period. FEV1, forced expiratory volume in 1 sec; MCh PC_20_ FEV1, the provocative concentration of methacholine causing a 20% fall in FEV1; SEM, standard error of the mean.

**Figure 3:**
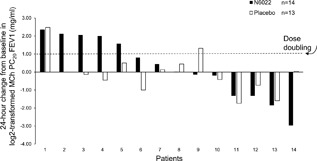
The change from baseline at 24 h in log2‐transformed MCh PC_20_ FEV1 after N6022 and after placebo for each patient is shown (with the exception of one patient who did not receive placebo). An MCh PC_20_ FEV1 change of one in log2‐transformed data represents a dose doubling. At 24 h, 5 of 14 patients (36%) had a dose doubling after N6022 and 2 of 13 patients (15%) had a dose doubling after placebo. MCh PC_20_ FEV1, the provocative concentration of methacholine causing a 20% fall in FEV1.

**Figure 4:**
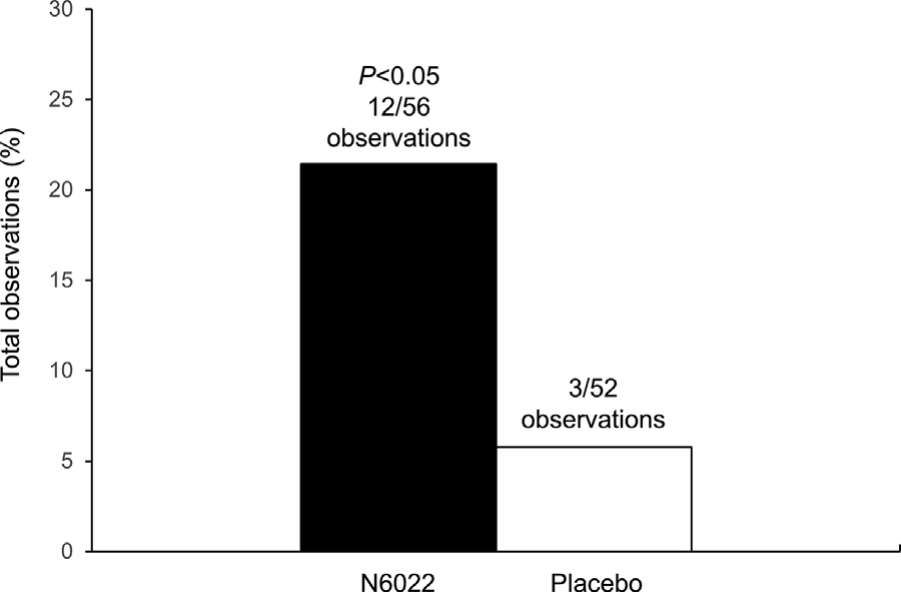
Percentage of total observations (at 8, 24, and 48 h and Day 7 combined) showing a two dose‐doubling increase in MCh PC_20_ FEV1 compared with baseline. During the 7‐day post‐treatment observation period, N6022 produced a significant increase in the percentage of observations of two dose‐doubling increases in MCh PC_20_ FEV1 compared with placebo. FEV_1_, forced expiratory volume in 1 sec; MCh PC_20_ FEV1, the provocative concentration of methacholine causing a 20% fall in FEV1.

The prespecified primary endpoint, MCh PC_20_FEV1 measured at 24 h after a single dose of N6022 5 mg, failed to show a statistically significant difference between placebo and N6022. However, exploratory analyses demonstrated statistically and clinically significant bronchoprotective effects of N6022 compared with placebo over the 7‐day post‐treatment observation period. At 24 h post‐dose the change in MCh PC_20_FEV1 was +1.48 mg/ml from a baseline of 1.34 mg/ml on N6022 vs −0.2 mg/ml from a baseline of 1.16 mg/ml on placebo (*P* = 0.49). The change from baseline averaged over the 7‐day post‐treatment observation period showed a significant effect with N6022 compared with placebo (mean change +0.82 mg/ml post‐N6022 vs −0.18 mg/ml post‐placebo, *P* = 0.023) (Fig. 2). Responders were defined as those patients with a dose doubling increase in the MCh PC_20_FEV1 compared with baseline within 24 h post‐treatment. The percentage of patients with a dose doubling in the MCh PC_20_ FEV1 at 24 h was 36% (5 of 14 patients) post‐N6022 compared with 15% (2 of 13 patients) after receiving placebo (Fig. 3). N6022 produced a significant increase in the percentage of two dose‐doubling increases in the MCh PC_20_FEV1 over the 7‐day post‐treatment observation period (21% vs 6%, *P *< 0.05, Fig. 4). Individual responses of each subject to placebo versus N6022 at 8, 24, 48 h and 7 days post‐treatment are provided in the Online Data Supplement, Figures S1–S4.

#### ECP levels

**Figure 5:**
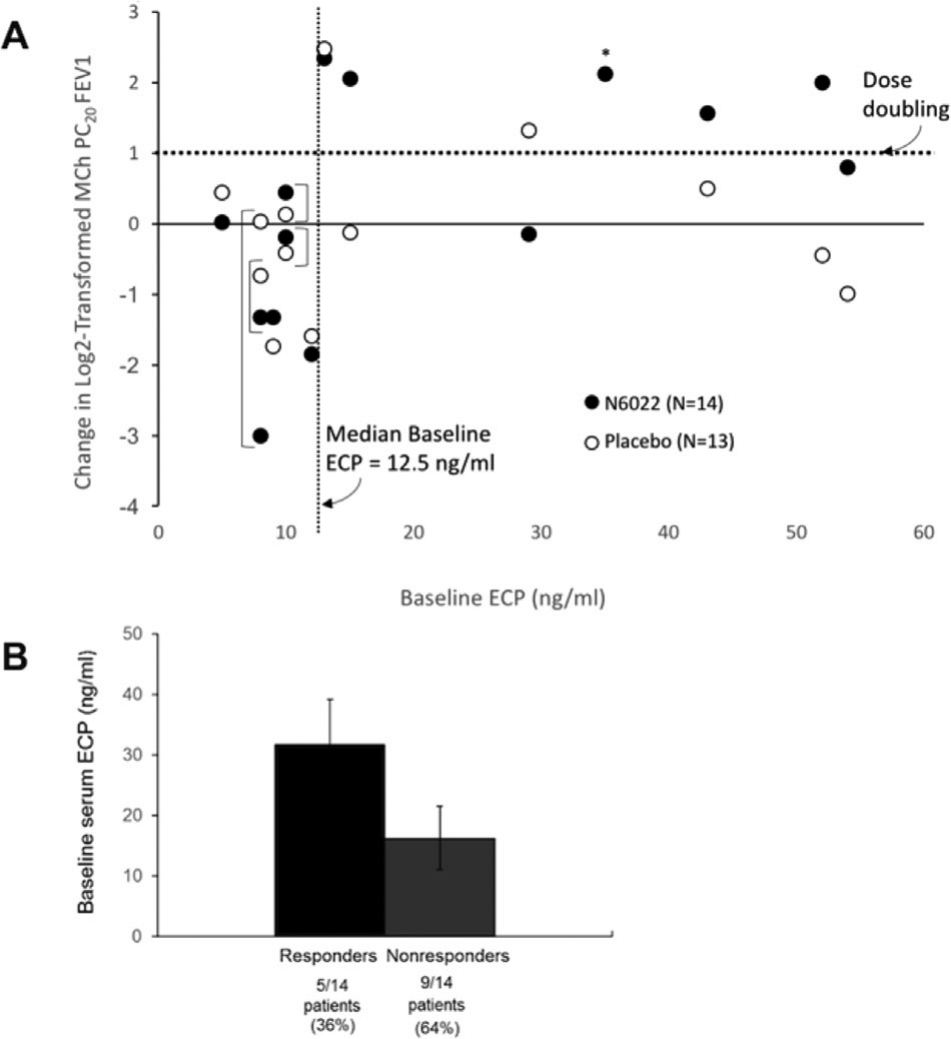
Investigation of baseline ECP levels and response. (A) Distribution of change from baseline at 24 h in log2‐transformed MCh PC_20_ FEV1 versus baseline ECP concentration. Each subject's response to N6022 (closed circle) and placebo (open circle) is provided per specific ECP level. For example, the patient with the lowest ECP level of 5 ng/ml (left side of figure) had a log2‐tranformed placebo change from baseline of 0.59 and an N6022 change from baseline of 0.02. The N6022 change from baseline of 2.12 marked with an asterix (*) is the patient with a baseline ECP of 35 ng/ml who withdrew from the study before placebo treatment. Individual patient responses with the same baseline ECP levels to 8 and 10 ng/ml are marked with brackets. An MCh PC_20_ FEV1 change of one in log2‐transformed data represents a dose doubling. (B) ECP Levels at baseline in responders vs non‐responders in the N6022 treatment period. Responders in this analysis were defined as those patients with a dose‐doubling increase in the MCh PC_20_ FEV1 compared with baseline at 24 h post‐treatment. ECP, eosinophilic cationic protein; FEV1, forced expiratory volume in 1 sec; MCh PC_20_ FEV1, the provocative concentration of methacholine causing a 20% fall in FEV1.

The correlation between the presence of inflammation at baseline and MCh PC_20_ FEV1 response was also evaluated. A trend toward higher baseline ECP levels was seen in patients who experienced a dose‐doubling increase in the MCh PC_20_FEV1 after N6022 compared with those who did not (Fig. 5). Similarly, a significantly higher baseline ECP level was seen in patients with a >50% dose increase in MCh PC_20_FEV1 after N6022 compared with those who had a lesser change in MCh PC_20_ FEV1 (ECP 35.3 vs 11.4 ng/ml, *P* = 0.005). All N6022 responders had a baseline ECP value greater than the median of 12.5 ng/ml. No effect of N6022 on serum ECP levels was observed. Patients with elevated ECP (≥32.5 ng/ml) at baseline did not show a significant difference from baseline at 8, 24, or 48 h after treatment with N6022.

#### Other exploratory endpoints

There was no change in the pre‐MCh challenge FEV1 with N6022 compared with placebo. There was no effect at 24 h of N6022 on sputum inflammatory cell counts (eosinophils, neutrophils, macrophages, and lymphocytes) and no significant changes in patient‐reported outcomes at day 7 using the ACQ‐7 were observed (Table 2). N6022 plasma clearance was rapid (individual estimates ranged from 12.6 to 44.1 L/h). The geometric mean (% coefficient of variation) value was 31.3 (32.5) L/h. The maximum N6022 plasma concentration (C_max_) was generally observed at the end of infusion (ranged from 154 to 768 ng/ml similar to a study in healthy subjects [http://ClinicalTrials.gov Identifier: NCT01147406]). The PK profile was not consistent with the peak effect of the drug at 24 h post‐dose in this study and in animal models of asthma. By that time point, plasma levels of N6022 were below or close to the limits of detection (0.5 ng/ml). Please see online supplement for additional information.

**Table 2:** Asthma control questionnaire composite score (FAS population)

	Treatment period	Day‐1 baseline, mean (SD)	Day 7, mean (SD)
ACQ composite score	N6022	1.01 (0.550)	0.99 (0.578)
	Placebo	0.91 (0.536)	0.75 (0.506)

The mean ACQ composite score at baseline was 1.01 ± 0.550 before N6022 treatment period and 0.91 ± 0.536 before placebo treatment period. The baseline ACQ composite score values were consistent with the definition of borderline adequate control. A slight decrease in mean ACQ composite score was observed at Day 7 following both treatment periods (decrease to 0.99 ± 0.578 and 0.75 ± 0.506 for the N6022 and placebo treatment periods, respectively). While a decrease in ACQ composite score suggests improvement of asthma control, the ACQ composite score decrease for either group did not reach the minimally clinically meaningful change of 0.5.

#### Safety

N6022 was well tolerated compared with placebo in patients with mild asthma (Table 3), and no safety concerns were observed. The percentage of patients experiencing ≥1 treatment‐emergent adverse event (TEAE) (an event occurring after administration of the first dose of N6022 or placebo) was slightly greater but not significantly different during the N6022 post‐treatment observation period than in the post‐placebo observation period (79% vs. 69%). Headache occurred more frequently after N6022 than after placebo (36% vs. 23%); all occurrences were considered either unrelated or unlikely to be related to N6022. The percentage of patients who experienced TEAEs involving the system organ class of Respiratory, Thoracic, and Mediastinal disorders was higher after N6022 than after placebo (29% vs. 8%), and all events were considered to be unrelated to study drug. These events were cough, nasal turbinate abnormality, oropharyngeal pain, pharyngeal erythema, allergic rhinitis, and respiratory fatigue in the N6022 group, and oropharyngeal pain in the placebo group. No consistent trends or clinically significant differences in clinical laboratory parameters, vital signs, or electrocardiogram findings were noted between baseline and the post‐treatment observation periods.

**Table 3:** Summary of adverse events by treatment period

	N6022 (*n *= 14)	Placebo (*n *= 13)
SAEs, *n*	0	0
TEAE, *n*	23	18
Patients with TEAEs, n (%)	11 (78.6%)	9 (69.2%)
Number of patients with TEAEs		
Mild	6 (42.9%)	4 (30.8%)
Moderate	5 (35.7%)	3 (23.1%)
Severe	0	2 (15.4%)
Patients with TEAEs according to relationship to N6022		
Unrelated	4 (28.6%)	3 (23.1%)
Unlikely to be related	3 (21.4%)	3 (23.1%)
Possibly related	4 (28.6%)	3 (23.1%)

SAE, serious adverse event; TEAE, treatment‐emergent adverse event.

### Mechanistic research

**Figure 6:**
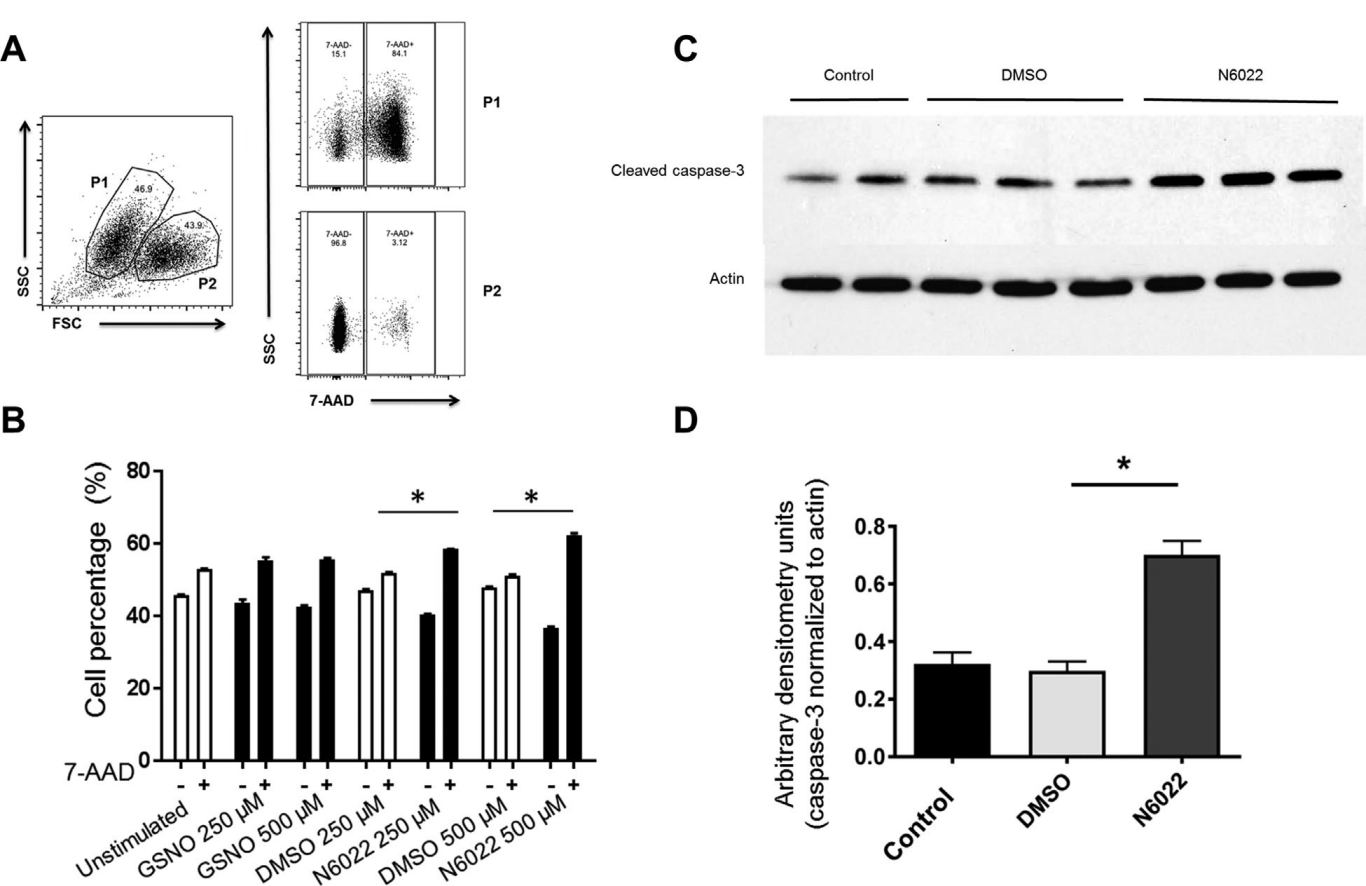
Increased eosinophil apoptosis observed in response to N6022 treatment. Flow cytometry analysis of eosinophil 7‐AAD to assess apoptosis in serum eosinophils extracted from IL‐5–transgenic mice. (A) The population with lower forward‐scattered light (FSC) was consistent with apoptotic eosinophils (P1) and the FSC‐high population was consistent with viable eosinophils (P2). (B) N6022 exhibited a dose‐dependent effect on the percentage of 7‐AAD‐positive cells. (C) Western blot analysis of cleaved caspase‐3 to determine apoptosis of serum eosinophils after treatment with N6022 or DMSO vehicle. β‐actin is shown as a loading control. (D) Densitometry performed on the western blot of the control and DMSO‐treated eosinophils vs N6022‐treated eosinophils. Caspase‐3 protein levels were normalized to actin and expressed as arbitrary densitometry units. * *P* <0.005. 7‐AAD, 7‐amino‐actinomycin D; DMSO, dimethyl sulfoxide; FSC, forward scatter; GSNO, S‐nitrosoglutathione; P, population; SSC, side scatter.

The mechanism of effect for N6022 was investigated using eosinophils isolated from IL‐5–transgenic mice. Percentage of cells stained with 7‐aminoactinomycin D (7‐AAD), a marker of cell apoptosis, were determined by FACS analysis of whole blood eosinophils from IL‐5 transgenic mice. Based on light‐scatter properties of the eosinophils, two cell populations were identified. The population with lower forward‐scattered light (FSC) (P1, Fig. 6A) was noted to be >80% 7‐AAD positive, consistent with apoptotic eosinophils. Conversely, the FSC‐high population (P2, Fig. 6A) was >80% 7‐AAD negative, consistent with viable eosinophils. We determined the relative percentage of 7‐AAD–positive from 7‐AAD–negative eosinophils dependent on treatment with vehicle (DMSO), GSNO, or N6022. We observed a significant increase in the eosinophil 7‐AAD–positive cells with N6022 treatment (Fig. 6B). N6022 exhibited a dose‐dependent effect, with the greatest percentage of the 7‐AAD–positive cells at the 500 μM dose, and a similar but less‐robust effect was noted with GSNO treatment (*P *< 0.0001 for both comparisons compared with DMSO controls). There was no effect noted with DMSO controls. Increased activation of cleaved caspase‐3 in N6022‐treated eosinophils was observed using western blot analysis indicating a greater level of eosinophilic apoptosis (Fig. 6C,D).

## Conclusion

Although the primary endpoint, a change from baseline in MCh PC20 FEV1 at 24 h, was not met in this exploratory early phase asthma clinical trial, a single‐IV dose of N6022 5 mg demonstrated a clinically significant reduction in AHR compared with placebo over 7 days. This was the first assessment of N6022 efficacy, safety, and tolerability conducted in fourteen patients with asthma (predominantly male), and individuals with mild asthma were selected because of their stable disease. Furthermore, due to their hyperresponsiveness to MCh (MCh PC_20_ FEV1 ≤8 mg/ml), this group offered a promising opportunity to test the bronchoprotective effects of N6022. Given the exploratory nature of the study and the small number of patients included, the improvements from baseline in the MCh concentration compared with placebo suggest potential efficacy for N6022 in asthma. These data are supported by an increase in the number of MCh dose doublings and two‐dose doublings on N6022 compared with placebo, during the post‐treatment observation period.

Although a change in baseline MCh pC20 FEV1 at 24 h was not met, it is not surprising as asthma is a highly heterogenous disease with high and low NO output. While it is well‐known that trials using the oral GSNOR inhibitor, cavosonstat, were not effective in the treatment of cystic fibrosis (CF), it is difficult to draw any conclusions from these trials as to how these drugs will perform in asthma. The lack of excess S‐nitrosylation in cystic fibrosis trials is likely the cause of failure in the CF patients and a potential explanation for the failure to meet the primary endpoint in this trial as only 4/14 subjects demonstrated a high degree of eosinophilic inflammation, a well recognized marker for increased endogenous NO production. Regardless, cavosonstat was well tolerated at doses that are predicted to exceed those required to inhibit GSNOR in the asthmatic airway and inflammatory cells and may be beneficial in severe asthma.

Furthermore, our preclinical study has demonstrated that preservation of GSNO levels is reported to be important for the regulation of airway tone and development of AHR in asthma 5. Previously, Marozkina et al. 21 observed marked heterogeneity in GSNOR activity in patients with severe and with non‐severe asthma. Thus, enhanced GSNOR activity is not a definitive feature in all patients with severe asthma and may impact just a subset of individuals. The development of next generation asthma therapies has repeatedly demonstrated that paired diagnostics, including genetic and phenotypic population stratification will likely be necessary to fully understand the potential of new therapies. Determining which types of patients are most likely to benefit from GSNOR inhibition may, in the future, provide an option for personalized asthma management 21. Our study population demonstrated significant variability in the allergic phenotype as assessed by serum ECP, a marker of allergic inflammation 22, and thus we explored the correlation between ECP and response to N6022. A categorical analysis of N6022 responders (those with a dose‐doubling in MCh PC_20_ FEV1 after N6022) and non‐responders showed that responders had approximately double the baseline ECP level compared with non‐responders. Consistent with that analysis, when ECP was treated as a continuous variable, all N6022 responders had a baseline ECP value greater than the median of 12.5 ng/ml. Previous studies of allergic asthma have used biomarkers including blood ECP, blood eosinophils, sputum ECP, and fractional excretion of nitric oxide (FeNO) to assess disease severity 23, 24, 25. Although a relationship has been observed between biomarkers 23, 24, 25. FeNO is considered one of the most practical, noninvasive method for diagnosis and monitoring of treatment response 26. The recent development of targeted asthma therapies directed at an type 2 inflammatory phenotype of severe asthma, such as anti‐interleukin (IL)‐5 therapy, anti‐IL‐4Ralpha, and CRTh2 antagonists, has benefited from investigation in populations selected based on FeNO and other measures of type 2 inflammation 27. The preliminary data presented here support the idea that GSNOR inhibition may be more beneficial in asthmatic patients with a type 2 inflammatory phenotype associated with eosinophils than those studied in this initial clinical trial and warrant further investigation of GSNOR inhibition in a selected population with more‐severe asthma. Of note, there is also a growing body of literature to suggest that genetic variants of GSNOR are associated with an increased risk of asthma. A study in Mexico City showed that genetic polymorphisms in GSNOR were associated with an increased risk of childhood asthma 13. Single‐locus analysis indicated that genotype variation in GSNOR was associated with a decreased response to beta‐agonist therapy in Africa‐American children with asthma 15. In a similar study, GSNOR and beta‐2 adrenergic receptor gene variants were associated with an increased risk of asthma and lower bronchodilator responsiveness in Puerto Rican children 12. Genetic polymorphisms were not studied in this proof of concept trial due to the limited number of subjects enrolled.

The supporting mechanistic research demonstrated an increase in eosinophil cell death by apoptosis with N6022. This may provide a suggested mechanism for the impact of N6022 in patients with more‐pronounced eosinophilia. Additional mechanisms of action for GSNOR inhibitors, suggested in other animal studies, include reductions in AHR, inflammatory mediators 15, 17 and mucus production 15. The greatest magnitude of effect was observed at a dose of N6022 500 μM, the highest nonclinical dose tested. Understandably, doses in laboratory‐based cell research do not translate to doses used in human studies but are useful as a proof of concept. Although in vitro studies using cell cultures can help investigate mechanisms, in vitro studies cannot account for PK and biotransformation and cannot be a basis for determining the most appropriate human doses 28. Doses of up to 10 mg/kg N6022 were effective in experimental murine models of asthma involving OVA sensitization and airway challenge to induce an allergic AHR 29. The dose of 5 mg N6022 was previously found to be safe and well tolerated in healthy subjects 19.

A limitation of this study is the lack of information on absolute serum eosinophil count. Degranulating eosinophils release both eosinophil peroxidase (EPO) and ECP, and although EPO is specific to eosinophils, ECP can also be secreted by neutrophils 30. Thus, the use of ECP to classify eosinophilia may present a limitation in this study. However, ECP was considered relevant for use here as it shows a relationship with asthma severity 23, 31 and has been reported to induce apoptosis via caspase‐3–like activity 32. The use of post‐hoc dichotomization of results is another potential limitation of the study [33]. Applying this statistical analysis methodology to a small study has the potential to produce unreliable results. As such, all data points were included in our final analyses.

Overall, in this first in man early phase exploratory trial, N6022 administered as a single IV dose of 5 mg was well tolerated in patients with mild asthma. No signal of treatment‐emergent toxicity related to N6022 was detected during the post‐treatment observation period; these findings suggest the absence of safety concerns that might prohibit further development of N6022 at the current dose. Taken together, these preliminary clinical and experimental data suggest that GSNOR inhibition may provide greater benefit to patients with a more‐severe type 2 inflammatory phenotype than those studied in this initial clinical trial. Thus, the next step of development would likely include testing of inhaled or oral N6022 in an enriched population of severe asthmatics stratified by type 2 asthma and/or genetic variants in the GSNOR gene.

### Clinical relevance

We showed that administration of an GSNOR inhibitor can be used safely in patients with mild asthma and resulted in bronchoprotection from nonspecific methacholine challenge over 7 days. In vitro, GSNOR inhibition induced eosinophil apoptosis. Patients with a higher baseline eosinophil cationic protein level experienced the greatest bronchoprotective effect. A high eosinophil cationic protein level may provide a means for identifying human asthmatics who are more likely to respond to GSNOR inhibitor therapy.

## Authors’ Contributions

Loretta G. Que was involved in the recruitment and evaluation of research study patients and interpretation of data. She was also involved in the design and interpretation of the preclinical studies and manuscript preparation. Loretta G. Que, Zhonghui Yang and Robert M. Tighe assisted with the design, performance, and interpretation of the eosinophil apoptosis studies. Njira L. Lugogo was involved in all the clinical research activities including recruitment and evaluation of research study patients and the review of study data and safety events. She also provided critical revision of the intellectual content in the manuscript. Rohit K. Katial was involved with the clinical trial design from inception. He was the principal investigator at National Jewish Health, Denver, reviewed data and contributed to manuscript preparation. Janice M. Troha was involved in the study design. David M. Rodman, Janice M. Troha, and Steven A. Shoemaker were involved in the review and analysis of the clinical efficacy and safety data. They also provided critical revision of the intellectual content in the manuscript. Monica Kraft was involved in the design of the study, recruitment and evaluation of research study patients, interpretation of data, and manuscript preparation.

## Conflict of Interest

J.M.T., D.M.R., and S.A.S. are employees of Nivalis Therapeutics, Inc., SAS

## Supporting information

Additional supporting information may be found in the online version of this article at the publisher's web‐site

**Figure S1‐S4**. The change from baseline at 24 hours in log2‐transformed MCh PC_20_ FEV1 after N6022 and after placebo for each patient at each time point is shown. Of note, patient 2 did not receive placebo.
**Table S1**. Summary of FEV1.
