# Supplementary material for: Effect of the S‐nitrosoglutathione reductase inhibitor N6022 on bronchial hyperreactivity in asthma
Source: Immun Inflamm Dis. 2018 Apr 11;6(2):322–31. doi: 10.1002/iid3.220 (PMC5946144; doi:10.1002/iid3.220)
Supplement: Supplementary file 1 — Figure S1‐S4. The change from baseline at 24 hours in log2‐transformed MCh PC20 FEV1 after N6022 and after placebo for each patient at each time point is shown. Of note, patient 2 did not receive placebo. Table S1. Summary of FEV1. [file IID3-6-322-s001.docx]

**Effect of the *S*-nitrosoglutathione reductase inhibitor N6022 on bronchial hyperreactivity in asthma**

**Authors:**

Loretta G. Que, Zhonghui Yang, Njira L. Lugogo, Rohit K. Katial, Steven A. Shoemaker, Janice M. Troha, David M. Rodman, Robert M. Tighe, Monica Kraft

**Online Data Supplement**

**Study population**

*Inclusion Criteria*

Patients who meet the following criteria will be considered eligible to participate in the clinical study:

1. Patient voluntarily agrees to participate in this study and signs an Institutional Review Board (IRB)-approved informed consent prior to performing any of the screening procedures and in the opinion of the principal investigator (PI) will comply with all the requirements of the study.

2. Males and females between 18 to 60 years of age, inclusive, at screening.

3. The following applies to female patients: a. Female patients must be of non-childbearing potential (surgically sterile [hysterectomy or bilateral tubal ligation] or post-menopausal ≥ 1 year with follicle stimulating hormone [FSH] > 40 U/L). Women receiving hormone replacement therapy (HRT) are eligible to enroll.

4. The following applies to male patients:

a. Agrees to use a condom and refrain from sperm donation from Day -1 until 30 days after dosing or

b. Has documentation of successful vasectomy.

5. Body mass index (BMI) between 18.5 and 35 kg/m2, inclusive, at screening.

6. Patient has a ≤ 5 pack years smoking history and nonsmoking for ≥ one year.

7. Documented history of mild bronchial asthma, first diagnosed at least 6 months prior to the initial screening visit and currently being treated only with intermittent short-acting beta-agonist therapy by inhalation.

8. Pre-bronchodilator FEV1 ≥ 70% of predicted at screening.

9. Sensitivity to methacholine with a provocation concentration of methacholine resulting in a 20% fall in FEV1 (PC20 methacholine) of ≤ 8 mg/ml at screening.

10. Demonstrated stable lung function during screening with ≤10% variability between two assessments of FEV1 taken at least 7 days apart at approximately the same time of day.

*Exclusion Criteria*

Patients who meet one or more of the following criteria will not be considered eligible to participate in the clinical study:

1. Past or present disease, which as judged by the investigator or medical monitor, may affect the outcome of this study. These diseases include, but are not limited to, cardiovascular disease, malignancy, gastrointestinal disease, hepatic disease, renal disease, hematological disease, neurological disease, endocrine disease or pulmonary disease (including but not confined to chronic bronchitis, emphysema, bronchiectasis or pulmonary fibrosis).

2. Clinically significant abnormalities, as judged by the investigator, in safety laboratory analysis during screening.

3. Patient has known history of hypertension or is hypertensive at screening. Hypertension at screening is defined as systolic blood pressure (BP) >150 mmHg or diastolic BP > 90mmHg. (May repeat measure within 30 minutes for possible inclusion)

4. Respiratory tract infection and/or exacerbation of asthma within 4 weeks prior to the first dose of study drug.

5. History of life-threatening asthma, defined as an asthma episode that required intubation and/or was associated with hypercapnia, respiratory arrest and/or hypoxic seizures.

6. Administration of steroids within 4 weeks of the screening visit. (Intermittent use of transdermal steroids is acceptable.)

7. Unable to abstain from other medications including non-steroidal anti-inflammatory drugs (NSAIDs), anti-histamines, anti-asthma, anti-rhinitis, or hay fever medication. The exceptions are fast acting beta-agonists taken less than daily, or acetaminophen ≤ 1000 mg/day is permitted except within 48 hours prior to Day -1 and hormone replacement therapy.

8. Unwilling to abstain from the following until study completion (comprising of both first and second treatment periods):

a. Smoking and other nicotine containing products

b. Consumption of grapefruit and grapefruit juice

c. Consumption of alcohol from 72 hours prior to inpatient admission and through inpatient stay

9. Positive blood test for ethanol at Day -1

10. The patient has a positive predose urine cotinine or urine drug screen

11. The patient has participated in a study with a new molecular entity during the previous 30 days prior to the first dosing day.

12. History of being unable to tolerate or complete MCh testing.

13. History of blood donation (500 mL) within 3 months of starting the clinical study.

14. The patient has tested positive for hepatitis C antibody or hepatitis B surface antigen.

15. The patient has tested positive for human immunodeficiency virus (HIV) antibodies.

**Study endpoints**

- The primary endpoint investigated whether a single dose of N6022 produced a significant bronchoprotective effect in patients with mild asthma, expressed as the MCh PC_20_ FEV1 at 24 hours post-dose compared with placebo
- Secondary endpoints were to:
  - Investigate whether N6022 produced a significant bronchoprotective effect (expressed as MCh PC_20_ FEV1) at 8 hours post-dose compared with placebo
  - Assess the safety and tolerability of single-dose administration of N6022 in patients with mild asthma based on adverse events (AEs), clinical evaluations, and laboratory assessments
- Exploratory endpoints investigated:
  - The bronchoprotective effect of N6022 over 7 days (expressed as MCh PC_20_ FEV1) compared with placebo
  - The potential effects of N6022 on serum eosinophil cationic protein (ECP) at 8, 24, and 48 hours post-dose compared with placebo
  - Whether N6022 decreased inflammatory cell counts in induced sputum at 24 hours post-dose compared with placebo
  - The bronchodilatory effects of N6022 using the FEV1 at 8, 24, and 48 hours post-dose and Day 7 compared with placebo
  - Respiratory symptoms using the Asthma Control Questionnaire (ACQ-7)
  - The pharmacokinetics of a single dose of N6022

**Randomization numbers**

- Prior to dosing on Day 1, patients were assigned a randomization number in accordance with the randomization code list generated by CPC Clinical Research prior to study start. The randomization code list was maintained and randomization was assigned by an unblinded party. The randomization codes were consecutive, four digit numbers beginning with 1001 and continuing to 1012 and were assigned consecutively as subjects were randomized at both sites.
- Once a randomization number was allocated to one patient, it could not be assigned to another patient. If a patient withdrew prematurely from the study and was replaced, under the direction of the Sponsor, a replacement number was assigned. Replacement patients were assigned to the same treatment as the discontinued patient. Replacement numbers were four-digit numbers starting with a leading 2. For example, if patient 1001 withdrew and was replaced, the patient number for the replacement patient would be 2001.

**Blinding and Breaking the Blind**

- The clinical study was performed in a double-blind manner. Once reconstituted, the drug product was identical in appearance to normal saline. Preparation of the drug product and the placebo (sterile normal saline) was performed by the research pharmacist so that the study blind was maintained at the pharmacy level.
- The study blind could not be broken except in a medical emergency (where knowledge of the IMP administered would affect the treatment of the emergency), with the exception noted below. In case of a medical emergency, the decision to break the blind was to be made on a case-by-case basis, at the discretion of the PI in collaboration with the Sponsor/Medical Monitor.
- The participants, care providers, investigators and the sponsor’s clinical research team responsible for the study were all blinded
- The first patient was enrolled on 26 March 2011 (screened on 14 march 2011) and the last follow-up visit was on 14 November 2011.

**Study assessments**

*Methacholine (MCh) Inhalation Challenge*

A MCh inhalation challenge was performed according to the procedures recommended by the American Thoracic Society (ATS) 2000 guidelines (E1), using the 2-minute tidal breathing technique. There was concordance of the MCh testing procedural method between sites. The test was terminated when a fall in FEV1 of at least 20% of the baseline value occurred, and the methacholine PC_20_ FEV1 was calculated. If the MCh testing was stopped before achieving a 20% fall in FEV1 or after reaching the highest dose of inhaled MCh of 16 mg/ml, the methacholine PC_20_ FEV1 was estimated using log-linear extrapolation from the FEV1 values at the last two MCh concentrations.

*Induced Sputum*

Induced sputum was performed according to the procedures recommended by the ATS 2005 criteria (E2). Induced sputum was performed before dosing on Day –1 and after MCh testing at 24 hours post-dose during each post-treatment observation period.

*Asthma Control Questionnaire*

The ACQ-7 was completed by the patient at Day –1 and Day 7 after dosing and was collected during both post-treatment observation periods.

*Safety and tolerability*

Safety was assessed by summarizing the incidence, relatedness, intensity, and type of treatment-emergent adverse events (TEAEs). AEs and serious adverse events (SAEs) that occurred after administration of the first dose of N6022 or placebo were considered treatment-emergent (TEAEs and TESAEs). Vital signs and twelve-lead electrocardiogram (ECG) data (observed and change from baseline). Infusion-site findings for each patient were listed and summarized by treatment, time point, and severity (score).

*Pharmacokinetic (PK) analysis*

Blood for PK analysis was collected on Day 1 before dosing, at the end of infusion (immediately after the post-infusion flush), and 0.167 (10 minutes) and 0.50 (30 minutes); 2, 4, 8, 12, 24 (Day 2) hours after the end of infusion. The individual patient concentration–time data were listed and displayed graphically on the linear and log scales. The concentration–time data was summarized descriptively in tabular and graphical formats (linear and log scales) by treatment. PK parameters were calculated using noncompartmental methods, with WinNonlin Version 5.1 or later.

**Statistical methods**

*Determination of sample size*

Given an estimated inter-patient MCh PC_20_ FEV1 standard deviation (SD) of 1.08 on the base-2 logarithm (log_2_) scale, a sample-size of 12 patients was determined to provide at least 80% power to detect a difference of 2 on the log_2_ scale, i.e., a quadrupling on the linear scale, between the treatment means at a two-sided 0.05 level of significance (E3).

*Statistical methods*

The full analysis set was defined as all patients who were randomized and who provided a MCh PC_20_ FEV1 at 24 hours post-dose during at least one of the two post-treatment observation periods. Patients were analyzed according to randomized treatment assignment. The safety population included all patients who received at least one dose of N6022 or placebo. The PK population included all patients who received active N6022 medication and provided at least one measureable concentration of N6022. MCh PC_20_ FEV1 and the change from baseline values for MCh PC_20_ FEV1 were summarized descriptively by treatment at each time point (n, mean, SD), without regard to period.

*Primary efficacy analysis*

The distribution of the primary endpoint, MCh PC_20_ FEV1 at 24 hours post-dose, was evaluated prior to unblinding for normality and, if the distribution was skewed, a log_2_ transformation was applied. The (transformed) MCh PC_20_ FEV1 data was then fitted to a mixed-effects model with fixed categorical effects for treatment, sequence, post-dosing time point and treatment-by-time-point interaction.

The baseline MCh PC_20_ FEV1 was specified as a continuous covariate, and a random effect for patient was specified to account for the correlation among the repeated observations taken on each patient. The denominator degrees of freedom of the F-test was specified using the Kenward-Roger approximation. The treatment-by-time-point interaction was retained in the model regardless of statistical significance, as the contrasts of interest are the differences in treatment effects at each post-dose time point.

Due to the lengthy washout period between observation periods, the carry-over effect of the first treatment sequence onto the second treatment sequence was assumed to be negligible. If the period effect was statistically significant (*P*<0.05), it was retained in the model and results were reported by period; otherwise, the final analysis model excluded the period effect. The N6022 post-treatment mean was compared to the placebo post-treatment mean at the 24-hours-post-dose time point using a contrast in the mixed model.

*Secondary efficacy analysis*

Comparisons of mean values of MCh PC_20_ FEV1 between N6022 and placebo at 8 hours, (secondary endpoint), 48 hours, and 7 days post-dose (exploratory endpoints) were analyzed by constructing contrasts in the mixed model used in the primary efficacy analysis above. All tests of hypotheses were two-sided with statistical significance defined as *P*≤0.05 with no adjustment for multiple comparisons.

All exploratory outcomes were to be summarized descriptively (n, mean, standard deviation, minimum, maximum) by treatment group at each time point without regard to period.

*Care and Use of Animals*

All experimental procedures in rodents were carried out in strict accordance with the recommendations in the Guide for the Care and Use of Laboratory Animals of the National Institutes of Health. The protocol and the facility housing the animals were approved by the Institutional Duke University Animal Care and Use Committee. Interleukin-5 (IL-5) transgenic mice (NJ.1638) on a C57BL/6 background strain were kindly provided by the late Dr. James J. Lee, PhD (Mayo Clinic Arizona) under a Materials Transfer Agreement. All mice were fed with standard mouse chow and housed in individually ventilated micro-isolator caging (up to 5 mice per cage) on a 12/12 light-dark cycle in a pathogen-free facility. Mice were fed PicoLab Rodent Diet 20 5053 mouse chow and given water ad libitum. Alpha-dry bedding was changed twice weekly. Nine mice were used in total. Blood was harvested from two-three naïve IL-5 transgenic mice (male and female), ages 2-5.5 months of age, to achieve a minimum of 10 million eosinophils per experimental protocol. All studies were performed in triplicate.

**Exploratory Endpoint: Bronchodilatory effects of N6022**

Pre-MCh challenge pulmonary function tests were performed at screening, pre-dose and at 2, 4, 6, 8, 24, 48 hours postdose and at Day 7 post-dose of each treatment period. Baseline was defined as the Visit 2 pre-dose FEV1 value of treatment Period 1. Observed FEV1 and %-predicted FEV1 values, and absolute change from baseline are summarized in Table S1. Overall, FEV1 did not show an appreciable change from baseline at 2, 4, 6, 8, 24, or 48 hours post-dose whether expressed as raw measures (L/s) or as % predicted. A slight increase in the FEV1 % predicted was observed in both N6022 and placebo treatment periods at the Day 7 post-dose visit (2.0% and 2.8% increase, respectively).

**Table S1: Summary of FEV1**

|  |  | **N6022 Treatment Period (n = 14)** | | **Placebo Treatment Period (n = 13)** | |
| --- | --- | --- | --- | --- | --- |
| **Time point** |  | **Observed** | **Change from baseline** | **Observed** | **Change from baseline** |
| Visit 2 pre-dose | N | 14 |  | 14 |  |
|  | Mean, L/s (SD) | 3.485 (0.5642) |  | 3.485 (0.5642) |  |
|  | Mean %-pred (SD)) | 83.5 (5.60) |  | 83.5 (5.60) |  |
|  | Min, Max | 2.38, 4.10 |  | 2.38, 4.10 |  |
| 2 hours post-dose | N | 14 | 14 | 13 | 13 |
|  | Mean, L/s (SD) | 3.370 (0.4756) | -0.056 (0.2463) | 3.438 (0.5302) | -0.050 (0.2552) |
|  | Mean %-pred. (SD) | 82.6 (7.62) | -0.9 (6.18) | 81.9 (7.16) | -1.0 (5.83) |
|  | Min, Max | 2.43, 4.03 - | 0.57, 0.25 | 2.54, 4.24 | -0.57, 0.28 |
| 4 hours post-dose | N | 14 | 14 | 13 | 13 |
|  | Mean, L/s (SD) | 3.426 (0.5306) | 0.000 (0.2318) | 3.480 (0.5823) | -0.008 (0.3308) |
|  | Mean %-pred. (SD) | 83.9 (6.37) | 0.4 (5.93) | 83.0 (9.61) | 0.1 (7.58) |
|  | Min, Max | 2.41, 4.17 | -0.36, 0.30 | 2.47, 4.27 | -0.86, 0.36 |
| 6 hours post-dose | N | 14 | 14 | 13 | 13 |
|  | Mean, L/s (SD) | 3.395 (0.4956) | -0.031 (0.2252) | 3.487 (0.5800) | -0.001 (0.3321) |
|  | Mean %-pred. (SD) | 83.2 (7.42) | -0.3 (6.09) | 83.2 (9.58) | 0.3 (7.86) |
|  | Min, Max | 2.37, 4.02 | -0.32, 0.38 | 2.53, 4.24 | -0.82, 0.42 |
| 8 hours post-dose | N | 14 | 14 | 13 | 13 |
|  | Mean, L/s (SD) | 3.431 (0.5530) | 0.004 (0.2495) | 3.505 (0.5267) | 0.017 (0.3114) |
|  | Mean %-pred. (SD) | 83.9 (8.45) | 0.4 (6.39) | 83.8 (8.99) | 0.9 (7.44) |
|  | Min, Max | 2.30, 4.24 | -0.34, 0.36 | 2.61, 4.14 | -0.73, .43 |
| 24 hours post-dose | N | 14 | 14 | 13 | 13 |
|  | Mean, L/s (SD) | 3.374 (0.5004) | -0.052 (0.2619) | 3.355 (0.5040) | -0.133 (0.3419) |
|  | Mean %-pred.(SD) | 82.6 (7.65) | -0.9 (6.32) | 80.1 (8.11) | -2.8 (8.02) |
|  | Min, Max | 2.47, 4.18 | -0.62, 0.35 | 2.51, 4.17 | -0.76, 0.35 |
| 48 hours post-dose | N | 14 | 14 | 13 | 13 |
|  | Mean, L/s (SD) | 3.384 (0.5141) | -0.043 (0.2685) | 3.494 (0.4637) | 0.006 (0.1953) |
|  | Mean %-pred. (SD) | 83.1 (8.30) | -0.4 (7.26) | 83.5 (4.31) | 0.5 (5.13) |
|  | Min, Max | 2.25, 4.24 | -0.43, 0.48 | 2.65, 4.18 | -0.29, 0.30 |
| Day 7 post-dose | N | 14 | 14 | 13 | 13 |
|  | Mean, L/s (SD) | 3.499 (0.5251) | 0.073 (0.2387) | 3.583 (0.4614) | 0.095 (0.3408) |
|  | Mean %-pred. (SD) | 85.5 (6.73) | 2.0 (6.04) | 85.7 (6.82) | 2.8 (8.37) |
|  | Min, Max | 2.58, 4.08 | -0.60 0.45 | 2.61, 4.25 | -0.71, 0.53 |

**Mechanistic research**

Apoptosis was evaluated for each group by analyzing the flow cytometry triplicate measures using an analysis of variance (ANOVA) with an independent factor for group and the corresponding interaction term, and stratification by 7AAD status (+/–). Contrasts using the two-way ANOVA interaction estimates were generated to compare the apoptosis for each group with the DMSO control group. A Bonferroni correction was used to control for false positives due to multiple group testing, which resulted in a critical alpha level of α=0.008 for determining statistically significant group differences from the unstimulated control group. For the densitometry performed on the western blot of the control and DMSO-treated eosinophils compared with N6022-treated eosinophils, statistical significance was tested using an unpaired t-test.

*Preparation of mouse eosinophils for flow cytometry (FACS) analysis*

Eosinophils were isolated from IL-5–transgenic mice. Briefly, IL-5 transgenic mice were sedated with an intraperitoneal injection of urethane 1 mg/g body weight; cardiac puncture was used to collect blood from IL-5–transgenic mouse and placed into EDTA tubes on ice. Red blood cells (RBCs) were lysed with 4 volumes of 1× RBC lysis buffer (eBioscience, San Diego, CA), washed in 5 ml of wash buffer (1× phosphate-buffered saline [PBS] with 0.1% bovine serum albumin [BSA]), then centrifuged at 1000 rpm for 5 minutes at 4°C. The cell pellet was washed again with 5 ml of wash buffer, counted and resuspended into 90 µl of MACS buffer for each 10 million cells. After a second wash, cells were re-suspended in 0.5 ml of MACS buffer. To ensure a purified eosinophil population, mouse CD45R and CD90.2 microBeads were added to the cell suspension according to the assay protocol (Miltenyi Biotec Inc., Auburn, CA) then loaded into the MACS LD columns. Flow-through cells were collected, centrifuged, and re-suspended in eosinophil culture medium (Roswell Park Memorial Institute [RPMI] 1640 with 10% fetal bovine serum [FBS]). Purified eosinophils were treated with 100, 250 and 500 µM GSNO or N6022, as well as respective vehicle controls with proportionate concentrations of dimethyl sulfoxide (DMSO) in the medium. Each treatment was performed in triplicate in 15-ml tubes with approximately 2 million purified eosinophils. Cells were incubated at 37 °C in a cell culture incubator for 20–24 hours. Following stimulation, cells were washed twice in cold PBS, re-suspended in staining solution with antibodies, and then stained for 15 minutes at room temperature in the dark. Following the incubation, flow cytometry was performed on the cells using a BD Canto II flow cytometer (BD Biosciences, San Diego, CA). The data was analyzed using FloJo software (version 10). The analysis was as follows: doublets were excluded using forward scatter-height [FSC-H] vs forward scatter-area [FSC-A]. Singlets were evaluated for light-scatter properties (FSC vs SSC). Based on light-scatter properties, the cells were defined for their expression of the viability dye 7-aminoactinomycin D (7-AAD). 7-AAD labeling was performed according to the manufacturer’s instructions (7-AAD, BD Biosciences, San Jose, CA). N=3 triplicates.

*Cleaved caspase-3 western blot*

For detection of cleaved caspase-3 (R&D Systems, Minneapolis, MN) and β-actin (Cell Signaling Technology, Danvers, MA), in serum eosinophils, blood from IL-5–transgenic mice was collected by cardiac puncture. After RBC lysis, cells were washed twice in wash buffer, and then resuspended in the eosinophil culture medium RPMI 1640 with 10% FBS. Approximately 5 million cells were used in each treatment group. Cells were harvested at 2 and 20 hours post-treatment, washed twice in ice-cold PBS, and lysed in cell lysis buffer (50 mM Tris-HCl, pH=7.5,138 mM NaCl, 5 mM ethylenediaminetetraacetic acid (EDTA), 0.2 mM diethylenetriaminepentaacetic acid (DTPA), 1% Triton X-100) with protease inhibitor cocktail (Fisher Scientific, Pittsburgh, PA) added before use. Approximately 3 µg of total protein was subjected to polyacrylamide gel electrophoresis (PAGE) gel separation and polyvinylidene difluoride (PVDF) membrane transfer (Bio-Rad Laboratories, Hercules, CA). Both cleaved caspase-3 antibody and β-actin antibody were used at 1:2,000-fold dilution.

**Figure Legend:**

Figure S1-4. The change from baseline at 24 hours in log2-transformed MCh PC_20_ FEV1 after N6022 and after placebo for each patient at each time point is shown. Of note, patient 2 did not receive placebo.

**References**

E1. American Thoracic Society. Guidelines for Methacholine and Exercise Challenge Testing-1999 *Am J Respir Crit Care Med* 2000;161:309‒329.

E2. Miller MR, Hankinson J, Brusasco V, *et al*. Standardisation of spirometry. *Eur Respir J* 2005;26(2):319–338.

E3. Inman MD, Hamilton AL, Kerstjens HA, Watson RM, O'Byrne PM. The utility of methacholine airway responsiveness measurements in evaluating anti-asthma drugs. *J Allergy Clin Immunol* 1998;101(3):342–348.


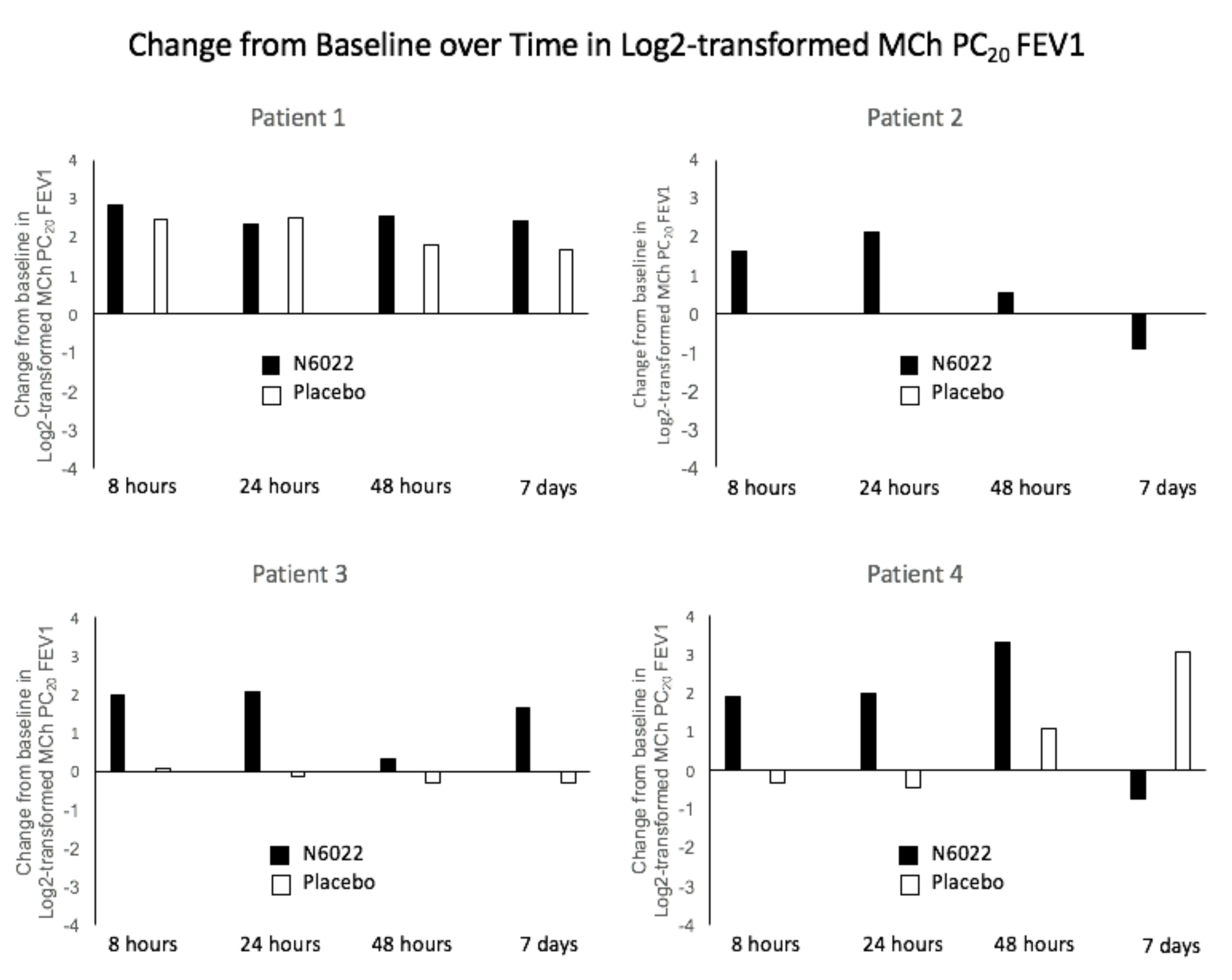


Figure S1.


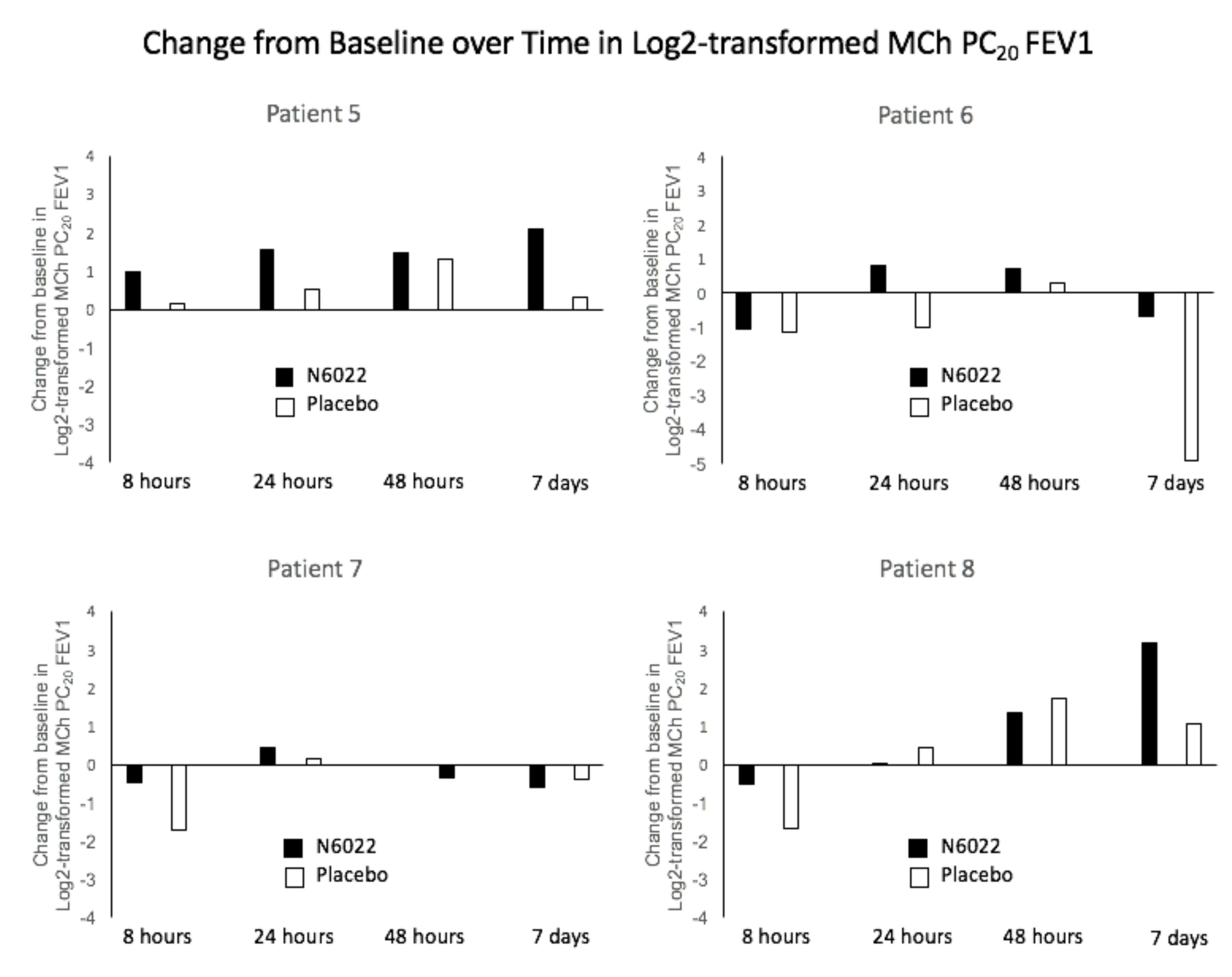


Figure S2.


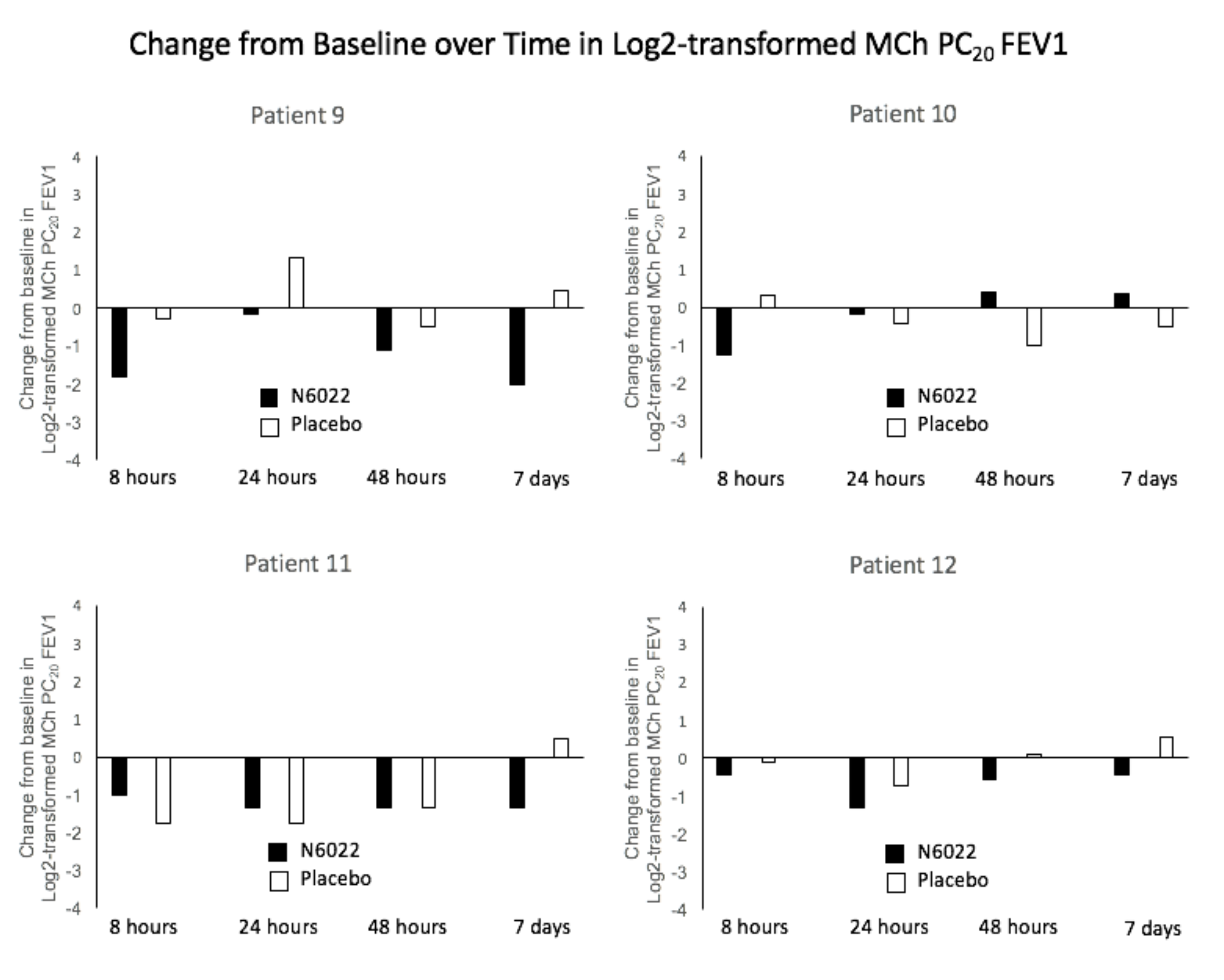


Figure S3.


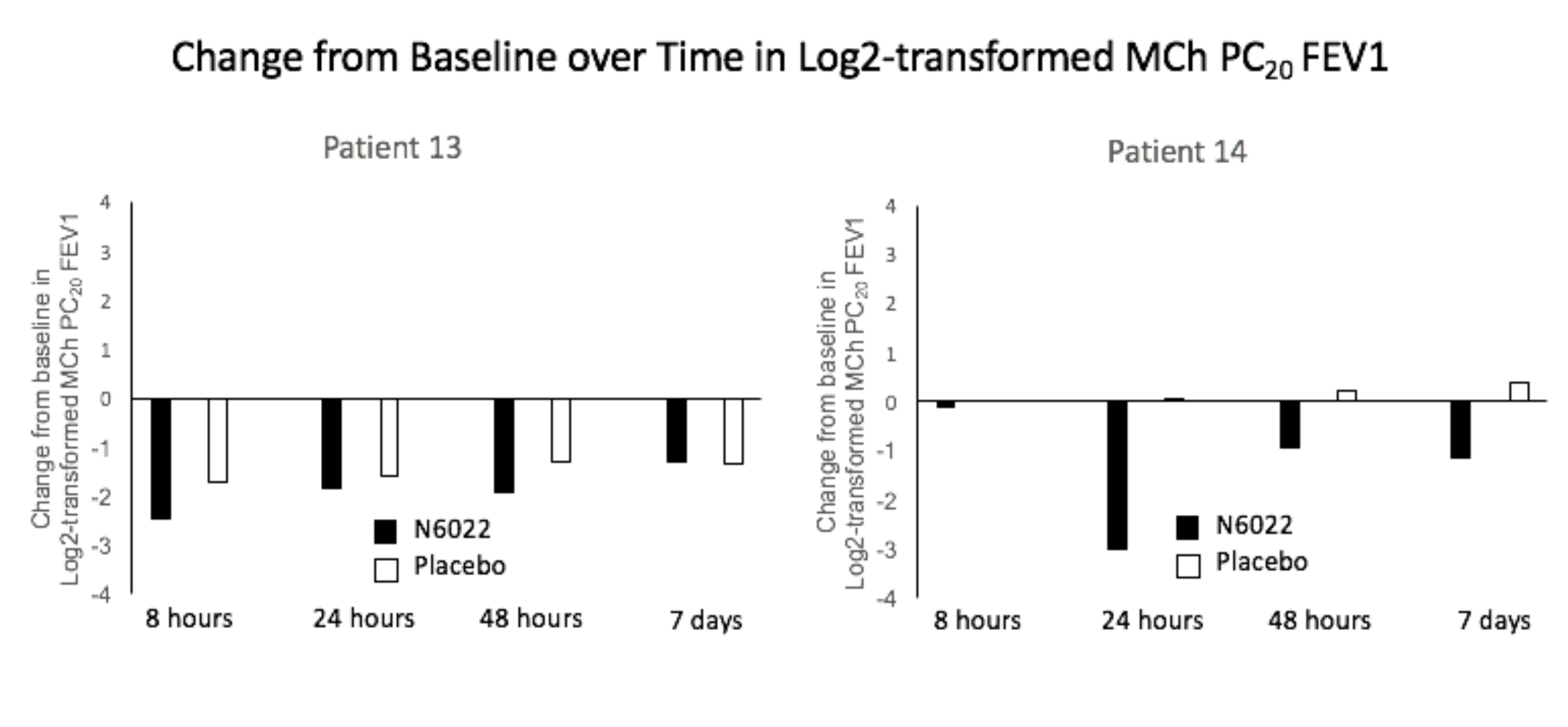


Figure S4.
